# Comparing Selection on *S. aureus* between Antimicrobial Peptides and Common Antibiotics

**DOI:** 10.1371/journal.pone.0076521

**Published:** 2013-10-18

**Authors:** Adam J. Dobson, Joanne Purves, Wojciech Kamysz, Jens Rolff

**Affiliations:** 1 Animal & Plant Sciences, University of Sheffield, Western Bank, Sheffield, United Kingdom; 2 Department of Entomology, Cornell University, Ithaca, New York, United States of America; 3 School of Molecular Medical Sciences, University of Nottingham, Nottingham, United Kingdom; 4 Faculty of Pharmacy, Gdansk University of Medicine, Gdansk, Poland; 5 Institute of Biology, Free University Berlin, Berlin, Germany; Rockefeller University, United States of America

## Abstract

With a diminishing number of effective antibiotics, there has been interest in developing antimicrobial peptides (AMPs) as drugs. However, any new drug faces potential bacterial resistance evolution. Here, we experimentally compare resistance evolution in *Staphylococcus aureus* selected by three AMPs (from mammals, amphibians and insects), a combination of two AMPs, and two antibiotics: the powerful last-resort vancomycin and the classic streptomycin. We find that resistance evolves readily against single AMPs and against streptomycin, with no detectable fitness cost. However the response to selection from our combination of AMPs led to extinction, in a fashion qualitatively similar to vancomycin. This is consistent with the hypothesis that simultaneous release of multiple AMPs during immune responses is a factor which constrains evolution of AMP resistant pathogens.

## Introduction

Environmental microbes readily evolve direct resistance to many powerful environmental stresses, whilst pathogenic bacteria avoid stress imposed by the immune system by evasion or subversion [1], [2]. Antimicrobial peptides (AMPs) are components of the immune system of multicellular organisms, and therefore are very prevalent in the environment, that usually kill microbes by selectively binding and disrupting prokaryotic cell membranes [3], [4]. AMPs are known to control both pathogenic [5], [6] and mutualistic [7] microbes. AMP resistance rapidly evolves at low cost *in vitro*: Resistance to pexiganan can evolve at low cost within just a few hundred generations in *Escherichia coli* and *Pseudomonas fuorescens* [8]. *Salmonella enterica* can evolve resistance to protamine and PR-39, and costs of resistance were either not observable or reversible by compensatory mutation [5], [9]. Nevertheless, susceptibility is variable in natural isolates [10]. AMP resistance thus presents a puzzling paradox: selection for resistance is widespread and it can arise at low cost, so why does variation persist?

AMP analogues have been proposed as next-generation antibiotics [11], [12]. Since active sites of AMPs are conserved, their putative therapeutic use stands to ‘arm the enemy’ with resistance to immune systems [12]. This concerning hypothesis has recently gained empirical support [13]. Understanding AMP resistance is therefore biomedically and evolutionarily interesting. To this end we must investigate costs and benefits of resistance to varied simulated immunological conditions.

Previous workers have suggested that natural AMP resistance is constrained by prohibitive intrinsic costs [3], however this is not consistent with *in vitro* data [5], [8], [9]. In immune responses multiple AMPs are usually transcribed after infection. Experiments in *Drosophila melanogaster* showed functional redundancy in AMPs, as fitness of flies mutant for AMP synthesis was dramatically reduced after infection, but restored by re-expression of just one AMP [6]. This suggests that the multiplicity of AMPs transcribed after infection serves a function other than just clearance of infection, which we hypothesized to be curtailing resistance to any single AMP. Biochemical studies have already demonstrated synergistic interactions between AMPs *in vitro* [14].

Here, we approach this principle by comparing the evolutionary response of the gram-positive bacterium *Staphylococcus aureus* (in which AMP resistance has already been experimentally evolved [13]) to *in vitro* selection from AMPs at standardised intensity, and investigate fitness consequences. Our study has three additional new features: (a) as AMPs are ubiquitous amongst animals we use AMPs from phylogenetically diverse taxa (mammals, amphibians, insects), all of which have been developed as antimicrobial drugs (b) we study the response to selection from two combined AMPs applied at the same intensity of selection as the parallel constituents; (c) we compare kinetics of AMP resistance evolution with antibiotic-selected treatment controls.

## Materials and Methods

We used *S. aureus* JLA 513 (from Simon Foster, Sheffield) which contains a chromosomal tetracycline resistance cassette that does not affect transcription or growth [15].

We used three AMPs and two conventional antibiotics as stressors. Pexiganan was kindly provided by Michael Zasloff, Georgetown University. Pexiganan was the first AMP to be developed for medical application [16] and kills bacteria by forming pores [17]. Melittin (Sigma-Aldrich M2272) is a well-studied membrane-permeabilizing peptide originating from honey bee venom [18], [19], [20]. Iseganan is a protegrin derived orginally from pig leucocytes [21]. Iseganan was synthesised by 9-fluorenylmethoxycarbonyl solid phase chemistry and purified on Kromasil sorbent, as previously [22], [23]. Pexiganan and melittin were also used in a 50∶50 combination (PGML): 1 µg ml^−1^ PGML contained 0.5 µg ml^−1^ pexiganan and 0.5 µg ml^−1^. Streptomycin (Sigma-Aldrich S9137) is an antibiotic derived from common environmental bacteria, so *S. aureus* is likely to have a history of association with it, reflected by ubiquitous streptomycin-resistant *S. aureus* [24]. By contrast *S. aureus* resistance to vancomycin (Sigma-Aldrich V1130) is less - albeit increasingly - common [1]. Since vancomycin resistance arises almost exclusively by horizontal gene transfer we predicted that vancomycin would be more robust to *S. aureus* resistance evolution in our study, but streptomycin resistance would be more facile. This allows us to qualitatively compare the responses AMP-selection to antibiotics that can (streptomycin) and cannot (vancomycin) be easily overcome by *S. aureus*, independent of antimicrobial mechanism.

Selection protocols followed [8] and allowed opportunities for standardized growth and evolution. All cultures were incubated at 30°C/120 rpm in SGM (standard growth medium: Müller-Hinton Broth [Sigma-Aldrich 70192], 5 µg ml^−1^ tetracycline, 5.6 µg ml^−1^ amphotericin-B). Before selection (day −10) *S. aureus* was inoculated into 5 ml SGM and grown for 24 h. 50 µl culture was passaged every 24 h for 10 days (day 0) - approximately 72 generations - to allow random mutation and accumulation of genetic diversity in our day 0 “ancestor” population.

Five parallel selection lines were established in each treatment at MIC_50_ (see below), alongside unselected controls. 5 µl samples (∼2.8×10^7^ colony forming units) of the ancestor culture were inoculated into 500 µl preparations of each treatment. 5 µl of 24 h cultures were daily passaged to fresh media. OD at 595 nm (OD_595_) of 100 µl of 24 h cultures were measured daily in a microtitre plate (Fig. S1). Remainders were cryofrozen in glycerol. Weekly, concentrations of treatment compounds were doubled (e.g. 8×MIC_50_ in week 4).

To check for contamination and to confirm the presence or extinction of *S. aureus,* cultures were diluted and plated bi-daily on LB 1.5% agar. Colonies displaying abnormal colour or morphology were re-plated on selective indicator medium (Mannitol Salt Phenol Red Agar [Sigma 63567-500G-F]) to verify that the cells were *S. aureus*. These protocols revealed no contamination through the course of the experiment. Extinction of populations was defined as two or more days with an OD equal to that of blank SGM, and no growth after plating onto LB 1.5% agar. Cultures were grown until extinction or the end of week 4 (Methods S1).

Minimum concentration of each AMP and antibiotic stressor required for total and 50% inhibition of growth (MIC and MIC_50_, respectively) and basic reproductive rate (r_0_) [2] were determined by dose-response assays in sterile 96-well microtitre plates, in a 2-fold dilution series of stressors (between 256 µg ml^−1^ to 0.125 µg ml^−1^, plus unsupplemented SGM for r_0_ estimation (Table S1)). To prepare cultures for assay, 50 µl of each culture was taken directly from the selection lines and grown in 5 ml SGM to late log-phase. 10 µl of culture was added to each well and OD_595_ was measured every hour for 6 hours, allowing exponential growth, which we take as our fitness measure.

Dose-response assays for MIC_50_ determination used bacteria from one culture of exponential-phase *S. aureus* JLA 513 (OD_595_ = 0.05). For assessment of MIC and r_0_ during the selection protocol, assays were performed on subcultures taken directly from the selection lines (50 µl selection line culture inoculated into 5 ml unsupplemented SGM) grown for 18 h and diluted 1∶10. Cultures showing aberrant growth or atypical starting OD were excluded from subsequent analysis *post hoc.*

r_0_ (basic reproductive/growth rate) was calculated for all cultures (i.e. 3 technical replicates in up to 11 different concentrations of stressor, plus unsupplemented media) in dose-response assays. r_0_s were calculated by logging the input OD data to linearise curves, and then taking the first derivative of a smoothed spline fitted to these data, representing the steepest point of the spline and therefore the maximum growth rate observed in our assays.

MIC_50_ was determined with the R package Grofit [25]. Bootstrapped smoothed splines were fitted to each growth curve and µ values were estimated. µ was used to construct dose-response curves, from which MIC (end weeks 1–4) and MIC_50_ (day −10) were estimated. Weekly fold-change in MIC of each culture was calculated. r_0_ was taken as median µ in unsupplemented media (See Methods S1[S3]). MIC was estimated as the first concentration in which there was no observable 6 h growth.

To calculate relative fitness indices, we took the mean of our measurements of r_0_ in unsupplemented medium of the three technical replicates of each of surviving populations per treatment per week. Since we expect some adaptation by all cultures to our protocols and experimental conditions, mean r_0_ values were of each population were normalised to the mean r_0_ of all populations in each respective week, to visualise how the fitness of each individual population changed.

## Results and Discussion

**Figure 1:**
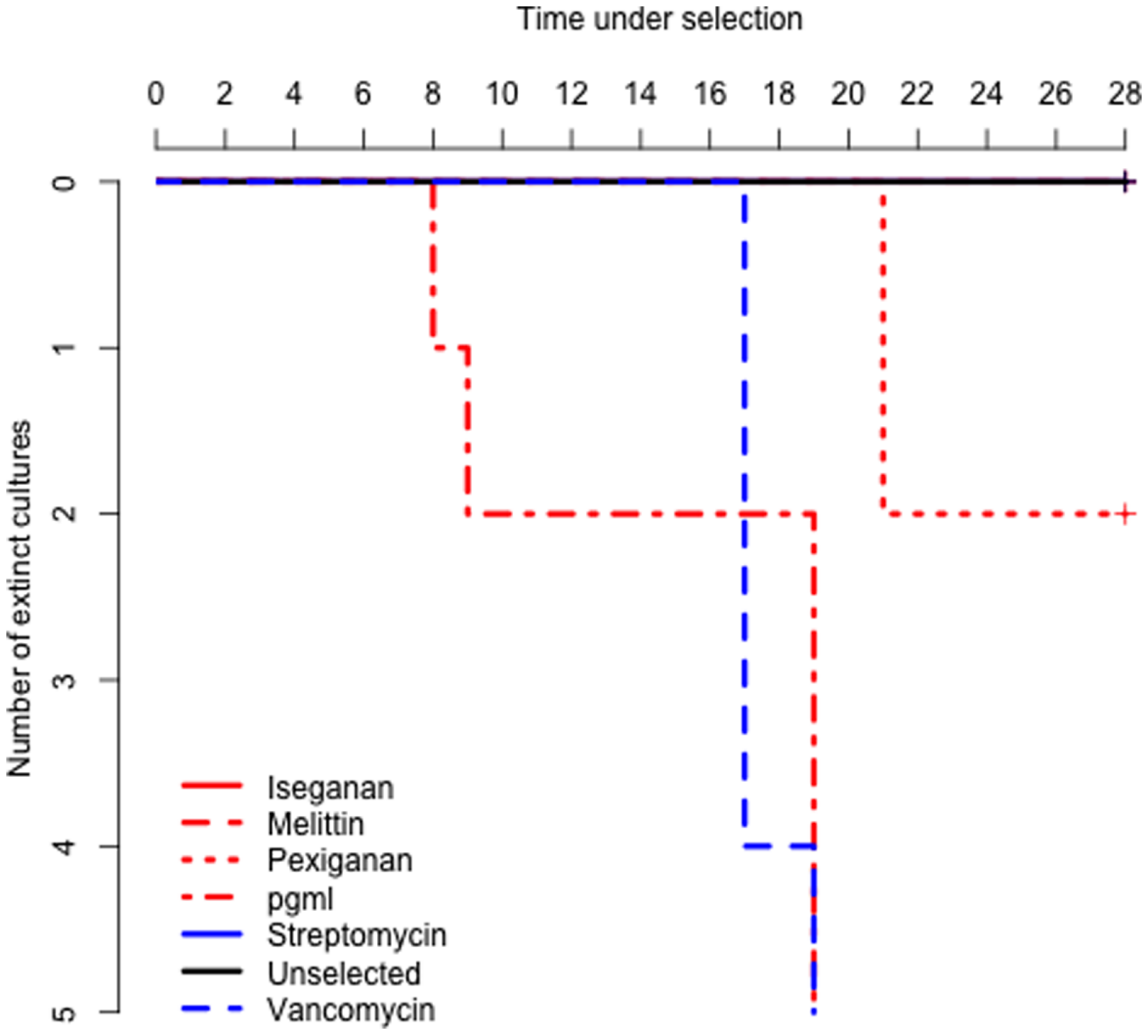
Population extinctions during AMP/antibiotic selection monitored over 28 days. Surviving lines are compressed into the top line of the figure.

All unselected procedural controls survived the duration of the experiment (Figure S1). PGML-selected cultures showed the earliest extinctions of all treatments (Figure 1) suggesting that simultaneous evolutionary responses to two stressors with different killing mechanisms was overwhelmingly challenging. Of the singly-selected cultures, iseganan- and melittin-selected cultures survived the duration of the experiment. Two pexiganan-selected cultures went extinct in week 4, when others were at low density (Methods S1). As predicted, viable streptomycin-selected cultures persisted for the duration of the experiment, and vancomycin-selected cultures went rapidly extinct in week 3. By this measure, melittin and iseganan presented the same evolutionary challenge as streptomycin. Crucially, our combined AMP (PGML) treatment led to more rapid extinction than any other treatment.

The evolution of resistance varied by treatment (Table 1). PGML-selected populations showed 1-4-fold greater resistance than the ancestor at the end of week 2, compared to 2-16-fold in mellitin- and 4-8-fold greater resistance in pexiganan-selected groups. Pexiganan-selected populations showed elevated MIC up to the end of week 2 (Methods S1). Iseganan- and melittin-selected populations showed steady increases in MIC throughout the course of the experiment. By the end of the experiment, iseganan-selected cultures were not inhibited by any concentration of iseganan up to 125 µl mg^−1^, in common with streptomycin-selected cultures. In contrast, vancomycin-selected bacteria showed stark increases in MIC over weeks 1 and 2, which is surprising given their extinction in the middle of week 3, highlighting that MIC assays do not always predict the robustness of an antimicrobial treatment over time (see Methods S1). Summarily, singly-administered AMPs can be overcome as easily as streptomycin, whereas their effect in combination is much more robust over time and comparable to vancomycin.

**Table 1:** Fold-change in MIC/population/week (median of 3 tests/combination), relative to ancestral population.

		Week			
Treatment	Population	1	2	3	4
Iseganan	1	8	>8	4	>16
	2	8	>8	4	>16
	3	8	8	**	>16
	4	8	8	4	>16
	5	8	8	4	>16
Melittin	1	4	4	8	32
	2	2	2	8	4
	3	2	4	8	4
	4	2	2	16	16
	5	2	16	*16*	*16*
Pexiganan	1	8	8	*l*	*l*
	2	8	8	*l*	*l*
	3	8	4	*l*	*l*
	4	4	8	*l*	*l*
	5	8	4	*l*	*l*
Pexiganan & melittin 50∶50	1	2	1	*e*	*e*
	2	2	**	*e*	*e*
	3	4	4	*e*	*e*
	4	2	4	*e*	*e*
	5	4	2	*e*	*e*
Vancomycin	1	>64	32	*e*	*e*
	2	>64	>64	*e*	*e*
	3	>64	8	*e*	*e*
	4	>64	32	*e*	*e*
	5	>64	32	*e*	*e*
Streptomycin	1	>8	>8	>16	>16
	2	>8	>8	>16	>16
	3	>8	>8	>16	>16
	4	>8	>8	>16	>16
	5	>8	>8	>16	>16

Zero observed inhibition is denoted by indication of MIC greater than the fold-change in MIC that would be inferred by MIC at the greatest concentration of stressor assayed.

** data excluded due to low OD upon inoculation into MIC assays.

l: low-density (pexiganan) and e: extinct cultures.

**Figure 2:**
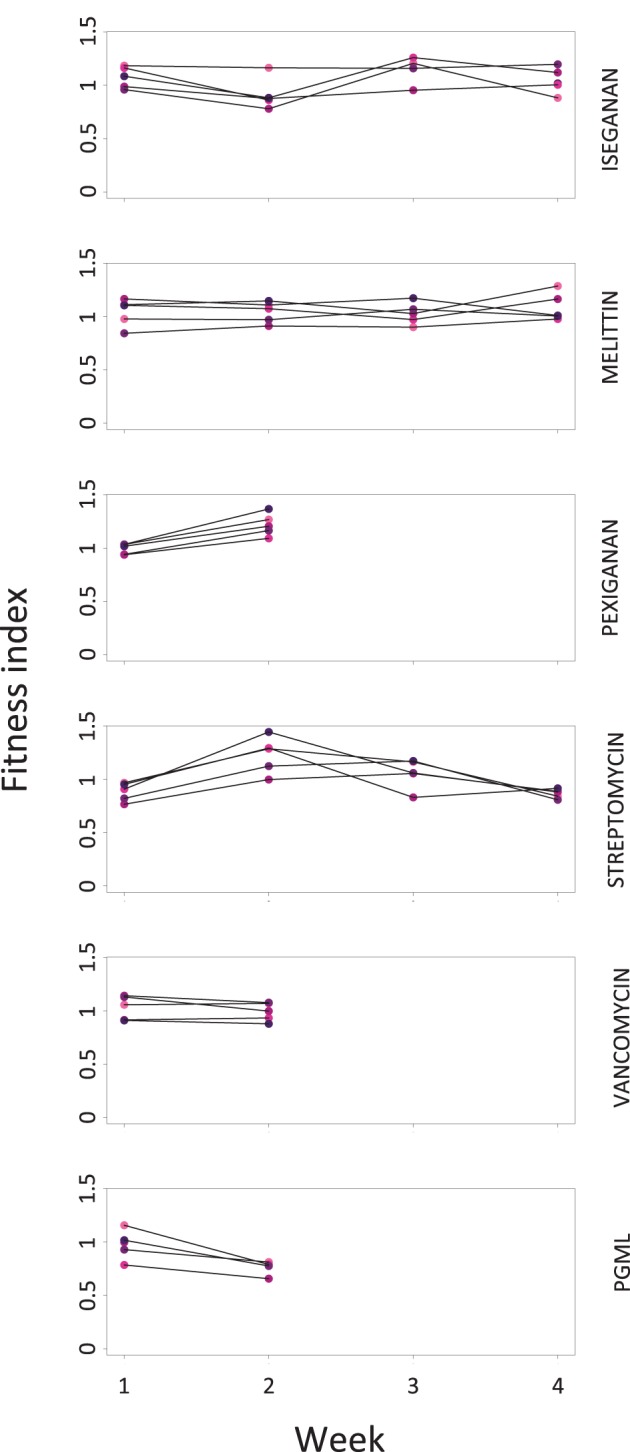
Weekly fitness indices of selected populations. Fitness indices were calculated as basic reproductive rate (R_0_) of each population (medians, n = 3) divided by average weekly R_0_. Pexiganan-selected cultures are excluded from weeks 3 and 4. Vancomycin and PGML-selected cultures were extinct by the end of week 3. 2-way ANOVA (week x treatment): F = 2.2, df = 14, p<0.01. At the end of week 2, only PGML showed significantly depressed fitness relative to the other populations (Tukey post-hoc comparisons: p_iseganan_ = 0.03, p_melittin_ = 0.02, p_pexiganan_ = 0.0005, p_streptomycin_ = 0.0003).

The three PGML-selected cultures that survived to the end of week 2 showed a significant depression of fitness relative to the three single AMPs and streptomycin at the end of week 2 (Figure 2). No other culture showed fitness costs. Since they did not become more resistant, we interpret the fitness effect observed in PGML-selected cultures as a lasting plastic (e.g. epigenetic) consequence of stress in the selection protocol, following failure to adapt to the stresses imposed by treatment, rather than an evolutionary cost. In other treatments that became more resistant to their stressors, we did not detect costs of resistance.

Our extinction, growth rate and resistance results are consistent with the principle that selection from combined AMPs is different to the sum of selection from the combination’s parts, since the response to selection from PGML was retarded relative to responses to the individual constituents. Technical limitations on the volume of iseganan that we were able to produce meant that we were unable to test this principle in a fully reciprocal design involving all possible combinations of our three AMPs. Further work is required to determine whether all AMPs are more robust to resistance evolution when administered or transcribed in combination. However we speculate that this is likely to be a general phenomenon, which is dependent on the nature of the functional interaction between the compounds, similar to effects of combinatorial administration on evolution of antibiotic resistance [26], [27], [28].

Consistent with previous studies [13], we were unable to detect a cost of resistance in terms of reproductive rate in our melittin- and iseganan-selected cultures. Pleiotropy may limit resistance when selection is applied from multiple AMPs: resistance to AMP A may increase susceptibility to AMP B, consistent with our hypothesis that simultaneous synthesis of multiple effectors by immune systems contributes to the constraint of *in vivo* evolution of immunoresistance.

Whilst we propose that multiple effectors constrain resistance to the immune system, this is likely to be only one of numerous interacting factors determining natural resistance. AMP resistance will also be a function of the costs and benefits of resistance during an infection. Simultaneously, holistic immunoresistance during pathogenesis is unlikely to be conferred by just AMP resistance. Benefits of AMP resistance will be low if the selection they apply is marginal relative to that applied by other effector systems e.g. phagocytes. It is additionally possible that bacteria have evolved mechanisms which are induced in response to exposure to AMPs, such as formation biofilms to limit exposure of a subset of cells. Having corroborated previous data on the costs of AMP resistance and expanded on it by considering costs of resistance to combined AMPs, our selected cultures now constitute a resource which can be used for a full economic assessment of costs and benefits of AMP resistance *in vivo*.

We have demonstrated that a combination of AMPs does not behave additively with respect to the selection imposed on *S. aureus* over ecological time, since the response to selection from two combined AMPs was not the same as the response to equivalent selection from the constituents. This response was qualitatively similar to that of the robust antibiotic vancomycin, whilst the constituents of the combination behaved similarly to streptomycin. We propose that such interactive effects are likely to be a factor to constrain the evolution of microbial resistance to AMPs in their natural immunological context.

## Supporting Information

Figure S1 **24 hr OD over the course of the experiment averaged for the treatments.** Optical Density (595 nm) of S. aureus cultures under weekly doubling selection from a range of antimicrobial stressors were measured daily, 24 hr after inoculaiton (n = 5 per treatment). Cultures showing OD_595_<0.05 are assumed dead and have been excluded from means calculation.
(TIFF)

Table S1 **Stressor concentrations per experiment.**
(DOCX)

Methods S1 **Daily OD, stressor concentrations, vancomycin extinction.**
(DOCX)
