# Supplementary figures and images for: Comparing Selection on S. aureus between Antimicrobial Peptides and Common Antibiotics
Source: PLoS One. 2013 Oct 18;8(10):e76521. doi: 10.1371/journal.pone.0076521 (PMC3799789; doi:10.1371/journal.pone.0076521)

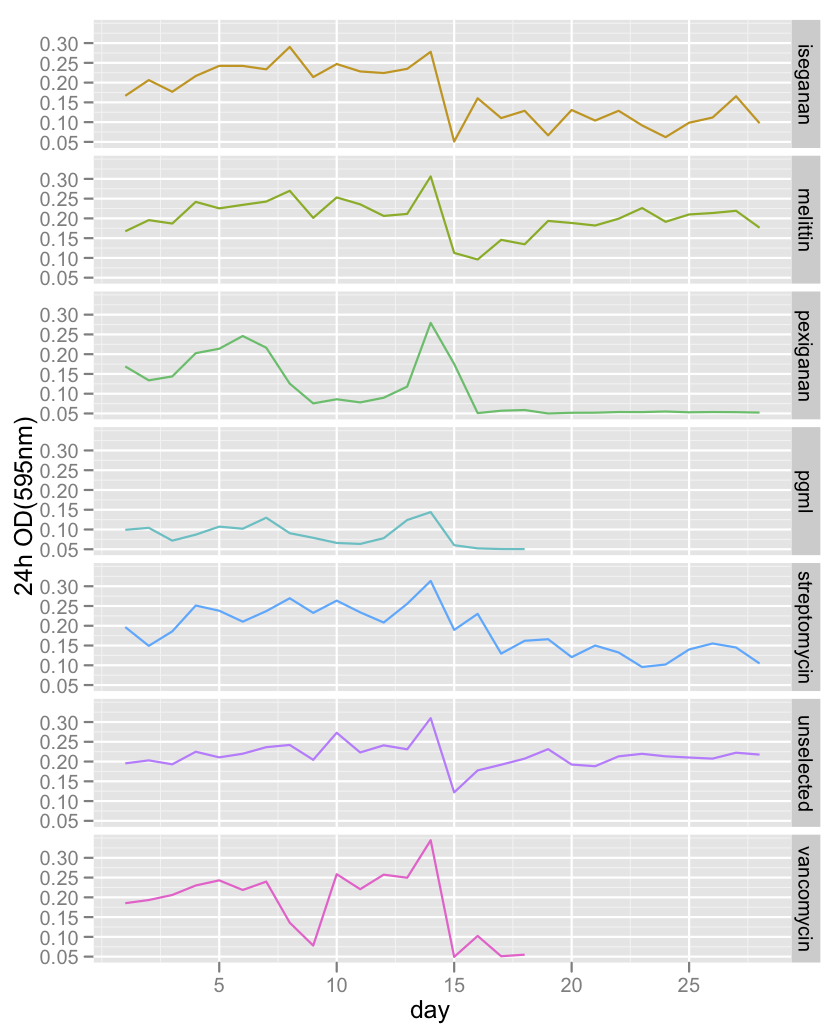

Supplement: Figure S1 — 24 hr OD over the course of the experiment averaged for the treatments. Optical Density (595 nm) of S. aureus cultures under weekly doubling selection from a range of antimicrobial stressors were measured daily, 24 hr after inoculaiton (n = 5 per treatment). Cultures showing OD595<0.05 are assumed dead and have been excluded from means calculation. (TIFF) [file pone.0076521.s001.tiff]
