# Supplementary material for: Comparing Selection on S. aureus between Antimicrobial Peptides and Common Antibiotics
Source: PLoS One. 2013 Oct 18;8(10):e76521. doi: 10.1371/journal.pone.0076521 (PMC3799789; doi:10.1371/journal.pone.0076521)
Supplement: Table S1 — Stressor concentrations per experiment. (DOCX) [file pone.0076521.s002.docx]

| **Table S1**. Stressor concentrations per experiment. | |
| --- | --- |
| **Experiment** | **Stressor range (2-fold dilutions, µg ml^-1^)** |
| MIC_50_ determination | 0, 0.125-64 |
| Weeks 1 and 2 dose-response | 0, 0.125-64 |
| Weeks 3 and 4 dose-response | 0, 0.125-128 |
|  |  |
