# Supplementary material for: Comparing Selection on S. aureus between Antimicrobial Peptides and Common Antibiotics
Source: PLoS One. 2013 Oct 18;8(10):e76521. doi: 10.1371/journal.pone.0076521 (PMC3799789; doi:10.1371/journal.pone.0076521)
Supplement: Methods S1 — Daily OD, stressor concentrations, vancomycin extinction. (DOCX) [file pone.0076521.s003.docx]

**S.1. Daily 24h OD.**

Optical density of each population was monitored daily 24h after establishment (Fig. S1). Incubator error meant that week-3 cultures had to be re-established from glycerol stocks. Glycerol stock equivalents of 5 µl of 24 h day 13 culture were inoculated into week 2 growth media and grown for 24h. Cultures were then pelleted and re-suspended in standard growth medium to within 0.02 of the 24h OD of the original day 14 cultures, to restore densities observed at the end of week 2. 5µl of each re-suspended culture was then sub-cultured into day 15 stressor conditions and the selection protocol resumed as per normal. The data presented from day 15+ are from these restarted cultures. Iseganan- and vancomycin-selected cultures grew slowly for the first day after re-establishment (Figure S1), but iseganan fully recovered. Vancomycin-selected cultures grew the following day then died, suggesting a problem of turnover (see S6). Further attempts to revive week 2 vancomycin-selected cultures from OD 0.1 and 0.15 led to the same failure of cultures (data not shown) mid-week three.

Our experimental design meant that MIC estimates could be influenced by variation in starting cell density (a function of population density in the selection protocol) large enough to prevent detectable exponential growth in the six hours after cultures were entered into the dose-response (MIC) assay. Therefore, since the pexiganan-selected bacteria were at strikingly low density in the selection protocol in weeks 3 and 4 (Figure S1), MIC estimates from these cultures are omitted in Table 1, despite the presence of viable cells in these cultures (Figure S1).

**S2. Stressor concentrations for dose-response assays**

Concentrations of stressors used in dose-response assays were varied according to the apparent resistance of the focal culture. Ranges of concentrations are detailed in Table S1.

**S3. Vancomycin resistance and extinction.**

Although our vancomycin-selected treatments showed no absolute growth inhibition in numerous iterations of the MIC assays during the selection experiment, we take the sudden and synchronous extinction of these populations at the beginning of week 3 as evidence that they were not highly vancomycin-resistant, as would be suggested by just the MIC assays. See S1 for further discussion with reference to restarting cultures from frozen stocks.

We propose that the low levels of growth shown by vancomycin-selected cultures at high concentrations of vancomycin in the dose-response assay was insufficient to overcome the daily turnover of cells imposed by our serial passage design, which approximates the natural turnover of cells in an infection: cells were growing, but too slowly to overcome the daily dilution imposed by passage into fresh media. This demonstrates that when testing the efficacy of antimicrobial compounds it is crucial to test their evolutionary stability with selection experiments, since simply assaying the presence or absence of bacterial growth in a the presence of compound does not predict the stability of resistance in the face of evolutionary change.
